# The Roles of Cultural Capital in Teacher–Student Interactions in China: A Qualitative Study of Students in Higher Vocational Colleges

**DOI:** 10.3390/bs13080690

**Published:** 2023-08-19

**Authors:** Junjun Huang, Meiyi Li, Shumin Wei, Zhen He

**Affiliations:** 1 Center for Educational Science and Technology, Beijing Normal University at Zhuhai, Zhuhai 519087, China; huang.junjun@icloud.com; 2 Faculty of Education, Beijing Normal University, Beijing 100875, China; 3 Faculty of Psychology and Educational Sciences, Ludwig Maximilian University of Munich, 80802 Munich, Germany

**Keywords:** cultural capital, higher vocational college, teacher–student interactions, China

## Abstract

The theory of cultural capital has long been applied to explain academic achievement. This qualitative study investigated first-year students in higher vocational colleges and, taking this low cultural capital group as an example, explored how cultural capital has affected their past teacher–student interactions. Participants described their experience of interacting with their teachers. We found that these students primarily experienced authoritative and laissez-faire teacher–student interactions. Students’ embodied and institutionalized cultural capital profoundly affected different aspects of the teacher–student interaction. In modern China, vocational and academic education are of the same status, so teachers should modify their evaluations based solely on academic achievement. When interacting with students, teachers should be more understanding and interact in a more student-centred way. Teacher training programmes should also be reformed, and training for teachers in general and vocational schools should be distinguished.

## 1. Introduction

The Chinese higher education system includes general universities, vocational universities, and higher vocational colleges. In 2021, students newly entering higher education institutions in China could be divided as follows: 41,380 (4%) in vocational universities, around 4.45 million (42.8%) in general universities, and 5.53 million (53.2%) in higher vocational colleges (Data come from the newest China Statistic Yearbook 2022: http://www.stats.gov.cn/sj/ndsj/2022/indexch.htm; accessed on 1 May 2023). Higher vocational college students are indisputably the most significant cohort of higher education learners in China, although they are considered the “losers” in academic education according to China’s college entrance exam [1].

Academic achievement is the main evaluation standard to distinguish between students who go to university or higher vocational colleges in China [2]. Cultural capital is a relevant factor in academic achievement. Indeed, Bourdieu has explained how students from privileged families have more cultural capital, which could help them adapt better to the school system [3,4]. Teachers have the most cultural capital in school, and they reward students who possess it; those rewards translate into better academic achievement [5,6]. Many previous studies have explored the relationship between cultural capital and academic achievement [7,8]. Students obtain cultural capital from their families, and this is valued by teachers at school. How teachers evaluate students with less cultural capital is not totally understood, however. Students with more cultural capital may communicate fluently with teachers, but what is the situation with students with less cultural capital?

The theory of cultural capital has been applied to explain many of the inequalities in education, which are mostly the result of differences in achievement [9]. It is well known that education should be diverse, and academic achievement should not be the only criterion of educational success. The teacher has the main role of valuing and rewarding the students in the school system, but the question remains: how do students perceive teachers’ preference for academic education? In particular, how do students with less cultural capital or poor academic achievement handle their interactions with teachers?

## 2. Literature Review

### 2.1. The Concept of Teacher–Student Interaction

Interaction between students and teachers is a key factor that could influence students’ academic performance and achievements [10]. Positive interaction is good not only for improving the quality of the classroom environment but also for improving students’ learning attitudes [11]. Traditional Chinese classes are normally dominated by the teachers, and student–teacher interactions are generally centred on the teacher’s instructional statements, questions, replies to student answers, statements about student behaviours, and so on [12]. Student–teacher interactions are even a microcosm of the social culture, Alexander [13] argued that classrooms reflect micro-cultures in their own right and are influenced by their local macro-cultures. Traditional Chinese cultural respect for the authority of elders represents a hierarchical element in student–teacher interactions [14]. While the teachers’ position of authority could explain how they interact with students, not much is known about how students perceive their interactions with teachers and how those interactions influence student learning through their various experiences with teachers. Ellis [15] defined teacher confirmation as “the transactional process by which teachers communicate to students that they are endorsed, recognized, and acknowledged as valuable, significant individuals”, which shows that the concept of the teacher in itself involves the meaning of interacting with students.

In this study, teacher–student interaction refers to all of the interactions and influences between teachers and students throughout the process of education. Teachers should interact with students as well as persuade them to participate and complete course objectives [16]. Although improved teacher–student interaction has been reported to have a strong relationship with students’ academic achievement, much less is known regarding students with poor academic achievement and their perception of teachers.

### 2.2. The Influence of Cultural Capital on Student–Teacher Interaction

A large number of previous studies indicate that cultural capital has a strong association with academic achievement [5,6,17]. Bourdieu pointed out that individuals with more cultural capital have more opportunities to obtain success through academic achievement [18]. Indeed, he believed that cultural capital has a deep relationship with social reproduction and that different social classes possess different levels of cultural capital. A previous study has suggested that social classes can be divided based on parents’ careers, as careers can, to some extent, reflect an individual’s cultural capital [19,20]. In this study, the middle class is defined as parents working as government officials, enterprise managers, private entrepreneurs, professionals, technocrats or academics; the working-class is defined as parents working as farmers, blue-collar workers or self-employed peddlers [20]. Middle-classes families normally have more cultural capital; for example, parents in middle-class families tend to have higher education certificates and more effective communication skills with teachers [21]. Working-class families, meanwhile, generally have different backgrounds: they lack cultural capital and tend to have a weak educational background or unstable careers [22]. Indeed, working-class parents tend to have less confidence in communicating with teachers as well as asking their children to respect and obey teachers at school [21].

Cultural capital has been divided into three forms: the embodied state (e.g., general cultural awareness, a taste of aesthetics); the objectified state (e.g., material books, paintings); and the institutionalized state (e.g., certificates of education, diplomas) [4]. Cultural capital is valuable in the educational field, and among the three cultural capital forms, the embodied state is the most relevant [23]. Objectified cultural capital, such as the trappings of elegant culture, can be considered to be ignorant, which is not conducive to improving the academic achievement in Chinese families [24]. In the educational field, as the goal of participating students is to meet teachers’ standards and move to the next level of schooling, students make use of their capital and especially the cultural capital they have received from families and prior experience, which results in academic success and positive feedback from their teachers [6]. At school, teachers value and reward certain cultural characteristics that are conveyed through daily interactions with students [6,23]. There is evidence in China that students from working-class families with less cultural capital can still obtain ultimate success in academic achievement due to their teachers’ encouragement and guidance [25]. Indeed, Bourdieu explained that an individual obtains cultural capital through a process of accumulation, with family as the starting place where individuals obtain their first cultural capital from parents, which is by school with teachers as the source [18,21,24]. Looking at the problems of education inequality in terms of cultural capital, social class or family background is not the only factor: we should also consider their interactions with teachers at school.

There are not many cases of working-class students having academically successful experiences with teachers. Other studies have discussed the negative interactions of working-class male students with their teachers based on the analysis of counter-school cultures [26]. In China, against the background of an applied counter-school culture, some students with less cultural capital refuse to conform at school, fight with teachers and engage in other negative interactions with teachers [24]. In general, most existing research has adopted qualitative and quantitative methods, with a primary focus on the relationship between institutionalized and embodied capital in family cultural capital, as well as the interaction between teachers and students [27,28] to reveal hidden educational injustice. Existing research has also tended to focus more on rural students [29] and “floating” children [30]. Less attention has been paid to vocational college students, who are the most typical representative group with low cultural capital in China, and even less attention has been paid to how students participate in and perceive teacher–student interactions. Examining how cultural capital affects teacher–student interactions and its influence on academic performance from a student perspective would be helpful to illuminate ways to help groups with weak cultural capital and to improve the current situation of educational injustice.

### 2.3. Application of Bourdieu’s Theory in the Chinese Context

The authors’ decision to use Bourdieu’s concept of cultural capital in this paper is a result of three main considerations. First, Bourdieu’s work has spread internationally over the past decades in the analysis of foreign schooling and inequality in the sociology of education [31,32]. Second, it has become a Chinese tradition for educational to use foreign theories to reanalyse Chinese educational problems by, for example, repurposing Western learning theories in Chinese classrooms [33]. Third, there is a growing body of research that has used Bourdieu’s theory to understand and analyse Chinese education. For example, Mu [34,35] and other researchers have integrated Bourdieu’s work into a theoretical analysis to examine inequality in contemporary Chinese education, including Shi and Li [36], with a focus on rural-to-urban migrant children’s habitus restructuring problem [35].

Bourdieu’s work has proved to be a useful tool to explore academic achievement and the problem of inequality in Chinese education [6,33]. Most of these studies have, however, ignored any exploration of vocational education. Although there are previous studies that have explored successful cases of Chinese working-class students entering general universities, those students have less family cultural capital and their teachers make up the deficit [24]. Many students are more likely to enter vocational schools since they failed in education competitions; they are likely to fall into the forced reproduction process of the weaker in the diploma society. Based on this reality, this study explored the following research questions:

(1) What kinds of teacher–student interactions did students experience before they entered into higher vocational colleges?
(2) What cultural capital do teachers and students use, and how is this cultural capital conveyed through daily interactions?

## 3. Methodology

A qualitative research methodology was applied in this study, as the purpose was to explore how the interactions between students and teachers influence students with poor academic achievement. It has been shown using a quantitative method that teachers’ cultural capital can influence students’ academic achievements [37]. In this study, the qualitative method allows the researchers to understand the meaning behind this phenomenon, as Guba and Lincoln [38] suggested that meanings could be constructed rather than discovered by qualitative research. This paper also discusses the issue from student perspective to understand how teachers’ interactions influence students’ learning attitudes and educational success or failure. From the students’ point of view, it is necessary to help teachers to reflect on themselves and adjust their teaching and communication activities in the future.

### 3.1. Setting and Participants

This study adopted the snowball sampling method, starting with a small number of initial contacts who fit the research criteria [39]. Nineteen students from higher vocational colleges were chosen to take part in this study, with students added until the interview data were saturated: the interviews with 16 participants were retained while 3 were deleted due to technical and personal problems. All of the interviewees were first-year college students. In China, a student’s choice of a vocational college or a general university is still based on their academic achievement, and higher vocational college students are the main potential interviewees [2]. To ensure the students have a vivid memory of their learning experiences before college, first-year students were chosen as the interview group. The participants were also asked about their family background. All of the participants came from working-class families with less cultural capital due to their parents’ poor institutionalized cultural capital—low educational credentials (only one father had a bachelor’s degree and more than half of the parents had no high school degree) and blue-collar jobs (factory workers and self-employed) [20]. All of the participants’ names in this paper are pseudonyms; their information is shown in Table 1.

### 3.2. Data Collection

A semi-structured interview outline was prepared in combination with the pre-interview, and the participants were invited to recall their learning experiences at school (The interview questions are displayed in the Supplementary Materials File S1). The data collection was completed in the form of one-on-one, semi-structured, in-depth interviews from December 2021 to June 2022, and each interview lasted around 1.5–2 h. When discussing students’ learning experiences, teachers were the most discussed topic. Based on vivid memories, the students’ learning experience in high school was the most frequently mentioned, followed by junior middle school and then primary school. Nobody discussed their kindergarten learning experience. The semi-structured interview was used as a supplementary interview method to make the interview more flexible. For example, when the interviewees mentioned their learning experiences with teachers, more detailed information was sought.

This study was conducted during the Chinese COVID-19 pandemic. Due to the strict quarantine policy, all of the interviews were conducted online with the use of Tencent VooV Meeting (The VooV meeting is an audio and video conference software, which has developed by Tecent. https://voovmeeting.com/; accessed on 1 May 2023). Online interviews allowed synchronous one-on-one interviews, with the interactions and reactions between interviewers and interviewees happening in real time [40]. However, the online interviews relied heavily on devices and networks, which meant that the interview process sometimes did not quite run smoothly.

### 3.3. Data Analysis

We analysed the obtained data from the semi-structured interviews using thematic analysis [41]. Using this approach, the researchers analysed the interview data flexibly and sought the potential meanings among participants’ experiences. NVivo was used to manage with interview contents. We followed a six-step data analysis process: (a) familiarization with the data: the authors transcribed and translated the interviews from voice into texts, from dialect to Mandarin and from Chinese into English; (b) generation of initial codes; (c) search for themes; (d) review of potential themes; (e) definition and naming of themes: to ensure the reliability of the coding and to triangulate the interview data, one author conducted her coding independently and all of the authors modified the data based on consensus—if there was, disagreement all of authors discussed the matter until a consensus was reached [42]; and (f) production of the report: the final coding scheme can be found in the findings section as Table 2 [43,44]. Numbers in parentheses represent the frequency of themes.

## 4. Findings

This study sought to understand the experience of teacher–student interaction in Chinese vocational schools and how cultural capital affects that interaction. Following the processes developed by Braun and Clarke [41], relevant themes were generated from students’ experiences with their teachers. The findings from this study can be divided into three main themes. There are two sub-themes related to the type of teacher–student interaction: (1) authoritative and (2) laissez-faire teacher–student interaction. There are three sub-themes related to how students’ embodied cultural capital affects the content and effectiveness of teacher–student interaction: (1) the transmission of academic cultural capital is the main content of interaction; (2) students’ low embodied cultural capital restricts interaction effectiveness; and (3) the acquisition of anti-school culture exacerbates teacher–student conflict. There are two sub-themes related to the ways differentiated institutionalized cultural capital affects the aim and content of teacher–student interaction: (1) teachers’ high institutional cultural capital affects the goals of teacher–student interaction; and (2) the transmission of institutional cultural capital affects the content of teacher–student interaction.

### 4.1. Types of Student–Teacher Interaction

#### 4.1.1. Authoritative Teacher–Student Interaction Dominates

Through the narratives of Chinese vocational school students, this study documented students’ perceptions of their teachers and their feeling of powerlessness with teachers’ authority. All of the interviewed students (*n* = 16) mentioned that they had experienced authoritative teacher–student interactions.

Teachers are authority figures in China, and this role has been influenced and developed by Chinese Confucianism. Teachers are either elders or seniors [45], and both parents and students feel that they should respect teachers. When students had misunderstandings with teachers, they were the less privileged group. They were confused about how to deal with such problems, in part due to the fact that the teacher figure was treated as the authority: teachers were an absolutely privileged group compared to students. One of the participants shared negative interactions with his teachers at school and explained that the interactions with a teacher were too passive: they had no choice but to obey the teacher’s arrangements when they had different opinions:

*When I was in junior middle school, I had bad academic achievement in chemistry and normally got bad grades… Once upon a time, when we had a chemistry class and we needed to do some chemical experiments, my chemistry teacher told us if we finished the experiment and passed his inspection we could leave the classroom… I was the first student who finished the experiment and I assisted my friend. When we finished, the chemistry teacher asked us to repeat the experiment in front of him, and we did it again. When we planned to leave, the chemistry teacher told us “If you guys leave today, never come back to my chemistry class again!”… I guessed that I threatened his authority at that moment, but he promised ahead, and I left when I fulfilled his requirement.*

(S3)

The participants were not encouraged to doubt their teacher’s words, and students could only follow their teachers’ rules. In other words, students were subordinate and teachers were dominant in teacher–student interactions. In the example above, the students followed the invisible interaction rules instead of arguing with or doubting their teacher. S3′s teacher questioned his experiments, and the teacher kept his face by breaking his own word. S3 also mentioned that perhaps he made his chemistry teacher had no face—after all, teachers in China were quite authoritative. However, the participants mentioned that they were always criticized by their teachers, which gave them no face and low self-esteem.

Most participants recalled that their academic achievements were not always good and that their teachers often criticized them, especially when they performed poorly or did not obey school rules. When asked about their feelings and being influenced by interactions with their teachers, participants mentioned that, of course, as students, they were a less privileged group and they did not know how to face their teachers. Students also remembered that their parents or other guardians always asked them to respect and not act out against teachers.

*I lived with my grandparents since I was young, because my parents worked in another city for many years… My grandfather had a primary school degree and my grandmother had no education degree; my parents had the same situation. My parents were not at home, so my grandparents were responsible for teaching me. When I began to have memories, I remembered that my grandparents told me to study every day and listen to the teachers’ words at school.*

(S2)

Based on the participants’ memories, some students noted that their parents resisted and seldom communicated with teachers. Instead of advising their children to communicate more with teachers, such parents told children just to listen to the teachers’ words.

*There were several times that my teacher in junior middle school wanted to do a home visit with my parents; however, my parents rejected all of them and told the teacher they were busy with work. Later, this teacher had no choice, but made a call and suggested my parents care more about my academic study.*

(S3)

*My father communicated with my teacher only once before I went to vocational college. He wanted my teacher to make some suggestions about my educational choice, to continue to study hard for an academic education or to choose vocational education. My father was not familiar with the different education styles, and he thought my teacher, as a worker in the education system, knew better than him.*

(S1)

#### 4.1.2. Laissez-Faire Teacher–Student Interaction

This study found that many students were in the marginal zone of school education, and the interaction with teachers was laissez-faire. When an interviewee was asked why he always skipped classes, he said that it was mainly due to the fact that he could not understand the class and the teacher was very lax. This revealed that some teachers were not willing to spend too much time and energy on students with poor grades.

*I couldn’t understand the teacher’s lecture. It’s also because the teacher’s management was relatively lax, so I felt bored staying in the class, so I skipped class.*

(S2)

One girl recalled her desperate attempted to improve her academic performance in junior high school to get her teacher’s attention, but her case reflected that, in some classes, only students with good academic performance could draw the attention of the teachers. Indeed, the teacher would often be more laissez-faire in the development of the students.

*Once I got full marks in math. I just wanted to get a compliment from the head teacher and the teacher’s affirmation that I made great progress. I worked hard for this… Since I was rebellious in the second year of junior high school, the class teacher felt that this girl seemed to be not good enough, and she couldn’t get into a good high school. Even if I could get into high school, I couldn’t get into a good key class. In the eyes of the head teacher, I didn’t seem to be good at anything. When I was in the third year of junior high school, I wanted to tell the teacher through my grades that I was not bad, I could do it.”*

(S13)

Laissez-faire teacher–student interactions were often the result of authoritative teacher–student interaction. Some students had the experience of being kicked out of the classroom since they did not study hard or since they violated class rules. When teachers treated students with poor grades or mistakes in this way, they did not educate or help students.

*When I was in high school, one night I checked messages on my mobile phone in my dormitory and the patrol teacher confiscated my mobile. I explained it to my headteacher the next day, and at first, he said that it was not a big problem and asked me to give up my mobile. Later, he asked me to go home and reflect. However, I could not leave the campus without the headteacher’s handwritten permission, and he refused to communicate with me and the guard. Thus, at that time, even though I was at school, I did not know what I could do. I was not allowed to enter my classroom and I was not allowed to leave the campus. The only thing I could do was wander around the campus.*

(S4)

As the exploration of laissez-faire interactions indicates, cultural capital has a profound effect on the process of teacher–student interaction.

### 4.2. Students’ Embodied Cultural Capital Affects the Content and Effectiveness of Teacher–Student Interaction

Embodied cultural capital refers to cultural products such as knowledge, upbringing, skills, tastes and sensibility acquired by actors through the family environment and school education, which become part of the individual’s spirit and body. The participants in this study were from higher vocational colleges, which indicates that they had failed in academic education in terms of the Chinese college entrance exams. Compared with students with good grades, academically poor students in the class are often labelled as having no knowledge and cultural accomplishment, thus becoming representatives of low embodied capital.

#### 4.2.1. Transmission of Academic Cultural Capital: The Main Content of Teacher–Student Interaction

Teacher-student interaction, as one of the main parts of the education process, is of great significance to students’ learning. This study found that the main content of teacher–student interaction was the transmission of academic cultural capital.

*As long as I had any problems, I would go to the teacher to talk directly… The main thing was that talking was a way of learning, what did I need to correct, or suggestions about what I should do and how to adjust. The most frequently asked questions were about learning methods.*

(S12)

In the descriptions of the interviewees, many incidents were about being punished by teachers for not studying hard, which became an important part of teacher–student interactions in their memories.

*Our high school teacher was our sports coach and also our head teacher. He was stricter in our studies. For example, every evening self-study, although he was a physical education teacher, but he teaching us mathematics, if we did not learn well, he would push us to run 800 metres, or run 400 metres. This kind of person cares more about our study and training, and is very responsible. He was very strict with us. If you wanted to be lazy during training, he would spank my ass with our training equipment. There was corporal punishment. He hoped that we could get all our grades in professional courses and cultural courses. He wanted me to go to undergraduate.*

(S1)

One participant described how older teachers at the school were rigid-minded and only concerned with learning, even making students spend less time in the shower. This case reflected that teachers pay too much attention to the improvement of academic performance, which lead to a negative state of teacher-student interaction.

*The class teacher in the second year of high school was a bit older, in his 50s. His thinking was a little bit more old-fashioned. He said that there is no need in life, just to do it so well, and focus on studying. Because we were often late for bathing, he said that in terms of bathing, it was winter at that time, and he said that there was no need to wash so many times a week: once a week was enough. I feel that my thinking is different from his, because young people like to be clean, and the frequency of bathing may be higher.*

(S2)

#### 4.2.2. Students’ Low Embodied Cultural Capital Restricts the Effect of Teacher–Student Interaction

According to the interviews, students with low embodied cultural capital were often unable to interact with teachers freely. They always lacked self-confidence in teacher–student interactions and had difficulty expressing themselves. Many students mentioned that they were introverted and unwilling, or had no courage to communicate with teachers in school. Although they hoped to grasp the teacher’s attention.

*When I was young, I was probably very introverted and didn’t talk much, including when I met some relatives during the Chinese New Year at home: as long as my parents didn’t make me say hello to uncle and aunt, I wouldn’t say hello. Then it was like kindergarten, and then in elementary school, I had less contact with my classmates, and I only played very well with my good friends.*

(S6)

*I might be afraid that I didn’t have a sense of existence. Most of the time, I hoped that the teacher would pay attention to some of them… Maybe it is my own problem. Sometimes I listened to my classmates chatting with the teacher more, they talked about some interesting things, and then I also wanted to have more exchanges with the teacher.*

(S5)

*My mother said that my grades in English were too bad, so I also went to tutoring. There was a foreign teacher over there. The foreign teacher would greet you warmly when he saw a new student, and then my temper was relatively slow, so I would be straight looking at him. Others would be very enthusiastic and respond quickly, but I was very slow… I was the most active after class, and I couldn’t wait to just shrink there in class and let the teacher not find me.*

(S5)

For students who were not good at communicating with teachers, the teachers also tended to neglect to establish contact with them. Teachers often paid less attention to these students, who had poor grades. Therefore, such students were told, “don’t cause trouble” or “don’t affect others’ learning”.

*That was, when the teacher asked me to serve as some class cadres, I would be a little happy because I felt that the teacher would pay attention to me. I always felt that the teacher didn’t pay much attention to me, because I was an average student in high school. It was not particularly good, and it was not particularly bad. Usually, there was no sense of presence. Awards were always given as a priority to good students.*

(S5)

#### 4.2.3. Acquisition of Anti-School Culture Exacerbates Teacher–Student Conflict

The anti-school culture is a cultural phenomenon that is contrary to the mainstream culture of the school. Although some students with poor grades were not expelled from school, they were excluded from the school system and they could easily form a group and be affected by the anti-school culture. A student described that the teacher arranged seats according to the test scores, so students with poor grades were arranged together. They formed a herd mentality of not learning.

*This was a fact that most people did not want to study. Those who studied and sat in the front, and those who did not study and sat in the back. As long as the teacher said, “don’t disturb them in class, everything else is fine.” Like most people, it might be a herd mentality.… Seats were assigned by the teacher. After he checked the grades, he put the good ones at the front of the classroom, and the bad ones next to or behind the classroom.*

(S7)

Another student recalled that he sat in the back row for some time as a result of poor grades, and developed the habit of copying homework with other students with poor grades.

*At that time, the ones in the back had a close relationship with some students in the next few rows. They chatted after class, and then they didn’t have to spend their spare time completing their homework. I would copy the homework assigned by the teacher, and I would take their test papers to look at, and then write it casually based on my own, and stop completing my homework so seriously.*

(S9)

Many students mentioned that they learned habits such as smoking from friends. If this habit was discovered, it often led to severe punishment from teachers. In the narratives of the interviewees, they mentioned that teachers would manage this student culture through simple and rude educational punishments. However, this resulted in the students not breaking the habit, but intensifying the conflict.

*When I was in junior high school, my friends taught me to smoke. At first, I thought it was funny, so I smoked with my friends. In that environment, I didn’t want to study anymore, and I confronted the class teacher every day. Then I asked for leave at noon, went to the bathroom for two hours at noon, and sometimes put on slippers and went to sleep in class… because the environment was like that, many people slept, and you felt that you were at a loss if you didn’t sleep.*

(S7)

*Once my teacher found out about my smoking and was notified. The head teacher knew about it the next day and asked me to go home and reflect on it for a few days. Then I stayed at home for a few days, rather decadent, just lying in bed. After a few days, my parents also knew about it and often criticized me. Finally, I wrote a review and handed it over to the class teacher. If I smoke in the future, I would be more careful, for fear of being caught.*

(S5)

### 4.3. Differentiated Institutionalized Cultural Capital Affects the Aim and Content of Teacher–Student Interaction

Diplomas are a typical form of institutionalized cultural capital. In the school system, there is a natural gap between the credentials of students and teachers. This study found that teachers’ high institutionalized cultural capital profoundly affected teacher–student interaction.

#### 4.3.1. Teachers’ High Institutional Cultural Capital Affects the Goals of Teacher–Student Interaction

Two students shared that they were encouraged by the stories of their teachers’ hard work during undergraduate studies and believed that they could also obtain better academic qualifications by studying hard.

*Our high school teacher repeated the course for three years, and he said that I would definitely be able to go to undergraduate.… You felt that his story was very inspirational. He repeated his studies in high school for three years. His mother died when he was in his third year of high school. It was a big blow to him, but he did not give up. At that time, his family conditions were very poor, and he was the kind who worked hard. His story was inspirational, you know we would train hard, study hard, and hope to pass an undergraduate exam.*

(S1)

*When I was in high school, my relationship with my teachers was still very harmonious. My class teacher was very kind, he teaches English, and although he would digress in his class, tell us some of his past stories and experiences, we should study hard and get it Some go to a higher level.*

(S9)

It can be seen that the goal of such teacher–student interactions was more dominated by teachers. Based on their own experience, teachers believed that it was their duty to help their students obtain better diplomas.

#### 4.3.2. Transmission of Institutional Cultural Capital Affects the Content of Teacher–Student Interaction

In addition to classroom interaction, the content of daily communication between teachers and students also focused on persuading students to work hard to get into an undergraduate course.

*After my cousin was admitted, my father found out that this school could also be used as an undergraduate, and he gradually had confidence in me. My father called the teacher to make an appointment to talk with him, asking me about my studies and professional courses, and Teacher Wu said that it was very promising. However, after talking together like this, my younger brother passed the exam in the same year, but I failed.*

(S1)

Especially teachers in the basic education stage, all hoped their students could be admitted to undergraduate schools in the future instead of entering vocational schools. Front-line teachers in middle and high schools did not, however, provide students with good examination guidance and career planning, and could not recommend suitable majors to students.

*As for why I chose this major, it was because the teacher said that there was school-enterprise cooperation, and he promoted it every day, sending me WeChat messages. There were many opportunities, and the school paid a lot of attention to it. Then there was a branch school-enterprise cooperation, which means that you can go to their companies for internships, and then choose your major without thinking too much.*

(S1)

From the perspective of the students, academic qualifications sometimes became an obstacle to teacher–student interaction. One student mentioned that he was not the same as a teacher with a postgraduate diploma.

*Although the teachers in the school were very nice, you could have a lot of communication with the teachers in learning. But to be honest, the teachers were all graduate students, and their world was the world you yearn for. But I was not sure whether I could pass the postgraduate entrance examination. Because I think that the teachers were all graduate students, and I might have nothing to talk about with them.*

(S11)

## 5. Discussion

This study explored how higher vocational college students, coming from working-class families, participated in teacher–student interactions. Whether the type of teacher–student interaction was authoritative or laissez-faire, there was still a lack of democratic interaction. That is to say, the teacher–student interaction impoverished by disparities cultural capital is an important reason for the poor performance of higher vocational students. When teachers are biased about cultural capital and offer too much academic education, it is unfair to students who may not have enough cultural capital. Students with poor cultural capital also cannot communicate with their teachers easily, which may then influence their academic achievement. With poor academic achievement, their teachers then care less about them, leading to a vicious circle. Students who can actively participate in teacher–student interaction generally have good academic performance [46]. Consistent with the conclusions of previous case studies on Chinese students with low cultural capital, positive interaction with teachers increased individual cultural capital, making up for the lack of family cultural capital and allowing students to achieve academic success [24].

This study found that cultural capital has a profound effect on teacher–student interactions, which is mainly reflected in two aspects: (1) students’ embodied cultural capital affects the content and results of teacher–student interaction, and (2) differentiated institutionalized cultural capital affects the goals and content of teacher–student interaction. Combined with Bourdieu’s classification of cultural capital types [4], this study found that objectified cultural capital is not the main type affecting teacher–student interaction. One of the reasons might be that most Chinese teachers do not value students’ objectified cultural capital (i.e., whether the student has a large collection of books or elegant artwork) in their interactions with students. This matches the prevailing culture in the Chinese school system, where objectified cultural capital may be seen as useless and detrimental to individual academic success [24]. Another important reason is that objectified cultural capital, tends to appear in the form of cultural commodities, which is more affected by family cultural capital. It was thus not deeply reflected in this study, because the participants came from working-class families with low cultural capital.

From the student’s point of view, having low cultural capital is an important reason for poor teacher–student interactions. On the one hand, due to their low academic performance, these students do not have equal opportunities in the interaction and attention from the teachers. On the other hand, because these students generally come from working-class families, they do not acquire the ability to communicate well with teachers at home. That is to say, the higher the family’s cultural capital, the more it can promote emotional and behavioural interaction between students and teachers, which is consistent with the conclusions of prior empirical studies [47]. Individuals who have been exposed to cultural capital and habitus generally from their birth, may feel more comfortable at school and more easily communicate with teachers [48]. The acquisition of anti-school culture would also intensify the conflict between teachers and students. These findings align with Willis’ study on counter-school culture, which revealed that working-class male students tend to have negative interactions with their teachers [26]. Such students were then more prone to resist the overall school culture. However, due to a lack of independent learning ability, they eventually wandered on the edge of class learning or even gave up school learning.

This study also demonstrated that teachers always showed prejudice against students who are mostly not high academic achievers, even when the students followed the rules in the classroom. In an increasingly serious diploma society, teachers acted as the agents of cultural capital hegemony and especially valued the type of cultural capital that appears as academic achievement. In 2022, the proportion of full-time teachers with a bachelor’s degree or above in China’s compulsory education stage reached 81.02% (Ministry of Education of the People’s Republic of China. The progress of the construction of the teachers in China in 2022. http://www.moe.gov.cn/fbh/live/2023/55167/sfcl/202303/t20230323_1052198.html; accessed on 1 May 2023). Teachers are the spokespersons of highly institutionalized cultural capital and the winners of previous school education. Educational action in schools is essentially an act of symbolic violence, in which teachers, as agents of the elite, assess and evaluate students according to the standards of elite culture [49,50]. Cultural capital guided the teachers’ interactions, which frustrated the learning of participants in this study. In line with the findings of Dumais, who contended that teachers possess the most cultural capital in the school system and reward students who have more cultural capital [5]. Some researchers have suggested that the family environment in childhood is not decisive for the acquisition of students’ cultural capital, and children of non-elite classes can acquire cultural capital from teachers [51,52]. However, this study found that students with low family cultural capital remained culturally disadvantaged in terms of teacher–student interaction, which would indirectly lead to a decline in academic performance. The low-cultural-capital student groups in this study did not obtain cultural capital in school in part because their teachers failed to deliver enough cultural capital due to prejudice.

## 6. Conclusions and Implications

The participants in this study were first-year students in Chinese higher vocational colleges. All of them performed poorly in the college entrance exams. This study investigated the past learning experiences of participants from working-class families through qualitative research methods. According to the interviews, “teacher” was the word that students most often mention. The teacher is quite an important factor in guiding students’ learning behaviours, and the teacher–student interaction is worth researching, especially from the student perspective. Interactions found in this study fell into two main types: authoritative and laissez-faire. In the Chinese context, teachers are still the centre of educational activities at school and are the privileged group: authority figures. From the perspective of cultural capital theory, we found that students’ embodied cultural capital affected the content and effectiveness of teacher–student interaction, while institutionalized cultural capital affected the aim and content of such interactions.

This study proposes a possible explanation for the microscopic process of the formation of educational inequality. Although some Chinese researchers have found that students who are disadvantaged themselves may form the underlying cultural capital [53], which will help students improve their competitiveness in the education process, this study conducted an in-depth analysis of how low-cultural-capital student groups understand and construct teacher–student interaction and how it affects their academic development through interviews with students in higher vocational colleges. This is a common situation for most students. Against the background of China’s social structure difference, promoting educational equity is an important issue for improving the level of teachers’ interaction in the field of education and the connection between compulsory and vocational education.

This study has implications for educational evaluation, teacher–student interactions and teacher training programmes in China. First, based on the Vocational Education Law of the People’s Republic of China 2022 (http://www.moe.gov.cn/jyb_sjzl/sjzl_zcfg/zcfg_jyfl/202204/t20220421_620064.html; accessed on 1 May 2023), vocational education and academic education have developed the same status in the Chinese education system: they are simply different types of education. Academic achievement should not be the only education of value for students. Teachers should observe and discover the characteristics of different students and guide them to choose the education type most suitable for them rather than guiding them only to choose academic education. Second, the teacher–student interaction styles should be modified. Teachers need to understand more of their students’ real thoughts rather than only asking students to follow what they are told. When interacting with students, teachers should treat students as equals instead of asserting their authority. The teacher-centred education style in China should also be modified. Students are the centre of educational activities, and they should be encouraged to express themselves when they have different opinions from teachers instead of resisting learning solely to resist the teachers. Third, in China’s teacher training programmes, there is no distinction between general school teachers and vocational school teachers, and all types of teachers are educated and trained at universities. This results in teachers’ preference for academic education. Teacher training programmes should thus be reformed. Teacher education should help teachers at the stage of compulsory education establish a view on the equality of general and vocational education, while improving teachers’ ability to teach according to their aptitude. In particular, for vocational school teachers, if they are biased towards academic education, the value of vocational education will decline and it will become an unfair education type for students. Vocational teachers should therefore be trained to obtain both theoretical knowledge and practical ability and, during their interactions with students, to guide the students to learn theoretical knowledge and practical skills.

This study did not discuss the students’ family backgrounds while focusing on the teacher interactions that influenced student learning, but an individual’s family background is the first place where individual obtains cultural capital [18]. The application of Bourdieu’s cultural capital theory into the problem of Chinese education inequality should also consider more family backgrounds. The topic of how parents’ cultural capital affects vocational college students’ learning would be worth further research. The present study was an in-depth investigation based on a small sample, so how cultural capital affects teacher–student interaction and academic performance deserves further quantitative research, which would help to understand its applicability in a broader group.

## Figures and Tables

**Table 1:** Participant information.

Name	Age	Gender	Parents’ Education	Parents’ Careers
S1	20	Male	Both: junior middle school	Self-employed
S2	19	Male	Junior middle school/no degree	Workers
S3	18	Male	Both: junior middle school	Workers
S4	19	Male	Primary school/high school	Workers
S5	19	Female	Bachelor/high school	Workers
S6	19	Female	Diploma/secondary education	Self-employed
S7	18	Male	Both: junior middle school	Self-employed
S8	19	Female	Both: secondary education	Worker and self-employed
S9	19	Female	High school/junior middle school	Workers
S10	18	Female	Both: high school	Workers
S11	18	Female	Both: junior middle school	Self-employed
S12	19	Female	Both: diploma	Workers
S13	17	Female	Both: junior middle school	Worker and self-employed
S14	18	Female	Junior middle school/high school	Self-employed and housewife
S15	18	Male	Both: high school	Workers
S16	19	Male	Both: junior middle school	Worker and housewife

**Table 2:** Coding scheme.

Main Themes	Sub-Themes (Frequency)	Description (Participants’ Parts of Experiences)
Type of teacher–student interaction	Authoritative teacher–student interaction (15)	Student was banned from class because he threatened the authority of the chemistry teacher. (S3)
		The teacher in charge of the junior high school strictly controlled, criticized to the point of discomfort, and did not respect personal privacy, so the student hated the teacher. (S9)
		Students were severely punished for disrespecting teachers and threatening the teachers’ authority because they completed their English workbooks in other classes. (S2)
	Laissez-faire teacher–student interaction (7)	Student could not understand the teacher’s lectures, and the teacher was too loose; the student felt bored and skipped class. (S2)
		The teacher told me to go home because I was on my phone, but the security guard wouldn’t let me leave the school. Caused me to hang out at school one day. (S4)
		The teacher felt that I was not promising and ignored me, which caused me to resist communicating with the teacher. (S3)
Students’ embodied cultural capital affects the content and effectiveness of teacher–student interaction	Transmission of academic cultural capital is the main content of teacher–student interaction (14)	Student criticized by the teacher for poor academic achievement. (S5)
		Older teachers are rigid in their thinking and focus only on academic education. (S2)
		If there is a problem while studying, I take the initiative to talk to the teacher. The interaction between teachers and students is mainly about academic education. (S12)
		The student was praised by the Chinese teacher for her well-written compositions, and they had good interactions, which promoted the improvement of her Chinese score. (S11)
	Students’ low embodied cultural capital restricts the effect of teacher–student interaction (10)	The student talked less, and she didn’t serve as class committee members, so she had less communication with teachers. (S6)
		I feel that the physics teacher was quite aggressive, so I did not dare communicate with him. (S5)
		Student didn’t like parents to build a good relationship with teachers through giving gifts. She worried about that it might cause criticism, so she reduced teacher-student interaction. (S8)
	Acquisition of anti-school culture exacerbates teacher–student conflict (8)	Taking up smoking with anti-school cultural capital triggered teachers to strictly control students, and students learned to confront teachers. (S4)
		Playing with students with poor academic achievement and developed the habit of copying homework. (S9)
		The teacher arranged the seats according to the exam results, and those with poor grades were arranged together, forming a herd mentality of not studying. (S7)
		Influenced by surrounding people and integrated into the “no-study” group. (S4)
		I’m confused at school, everyone else is studying, and I don’t know what to do in the dormitory. (S4)
Differentiated institutionalized cultural capital affects the aim and content of teacher–student interaction	Teachers’ high institutional cultural capital affects the goals of teacher–student interaction (4)	High school teachers use their own experience to encourage students to study hard and reach a higher educational level. (S9)
		The teacher took the college entrance examination three times to “succeed” and hoped that his student would also go to university. (S1)
		Teachers and parents want students to try again for the college entrance exam, but he failed at first. (S1)
	Transmission of institutional cultural capital affects the content of teacher–student interaction (4)	The teacher described the wonderful life of “going to university” to the students. (S1)
		The teacher did not give students a good career plan and recommended unsuitable majors. (S1)
		I felt that my teachers had a postgraduate degree, but I might not be able to pass the postgraduate exam, so I avoid communication with them. (S5)

## Data Availability

Data (qualitative materials including the interview transcripts) sharing is not applicable to this study due to protecting the participants’ privacy.

## Supplementary Materials

The following supporting information can be downloaded at: https://www.mdpi.com/article/10.3390/bs13080690/s1, File S1: Main interview questions.

## References

[1] Wang Q. (2013). China needs workers more than academics. Nature.

[2] Wang A., Guo D. (2019). Technical and vocational education in China: Enrolment and socioeconomic status. J. Vocat. Educ. Train..

[3] Bourdieu P., Brown R. (2018). Cultural reproduction and social reproduction. Knowledge, Education, and Cultural Change.

[4] Bourdieu P., Granovetter M., Swedberg R., Press W. (2018). The forms of capital. The Sociology of Economic Life.

[5] Dumais S.A. (2002). Cultural capital, gender, and school success: The role of habitus. Soc. Educ..

[6] Gaddis S.M. (2013). The influence of habitus in the relationship between cultural capital and academic achievement. Soc. Sci. Res..

[7] Byun S.Y., Schofer E., Kim K.K. (2012). Revisiting the role of cultural capital in east Asian educational systems: The case of South Korea. Soc. Educ..

[8] Roksa J., Potter D. (2011). Parenting and academic achievement: Intergenerational transmission of educational advantage. Soc. Educ..

[9] Barone C. (2006). Cultural capital, ambition and the explanation of inequalities in learning outcomes: A comparative analysis. Sociology.

[10] McGrath K.F., Van Bergen P. (2015). Who, when, why and to what end? Students at risk of negative student-teacher relationships and their outcomes. Educ. Res. Rev..

[11] Cipriano C., Barnes T.N., Kolev L., Rivers S., Brackett M. (2019). Validating the emotion-focused interactions scale for teacher-student interactions. Learn. Environ. Res..

[12] Wang S., Rubie-Davies C.M., Meissel K. (2019). Instructional practices and classroom interactions of high and low expectation teachers in China. Soc. Psychol. Educ..

[13] Alexander R. (2000). Culture and Pedagogy.

[14] Shengnan L., Hallinger P. (2021). Unpacking the effects of culture on school leadership and teacher learning in China. Educ. Manag. Adm. Leadersh..

[15] Ellis K. (2000). Perceived teacher confirmation. The development and validation of an instrument and two studies of the relationship to cognitive and affective learning. Human Commun. Res..

[16] Turman P.D., Schrodt P. (2006). Student perceptions of teacher power as a function of perceived teacher confirmation. Commun. Educ..

[17] Edgerton J.D., Roberts L.W., Peter T. (2013). Disparities in academic achievement: Assessing the role of habitus and practice. Soc. Indic. Res..

[18] Bourdieu P., Wacquant L.J. (1992). An Invitation to Reflexive Sociology.

[19] Weininger E.B., Lareau A. (2003). Translating Bourdieu into the American context: The question of social class and family-school relations. Poetics.

[20] Sheng X. (2017). Cultural capital, family background and education: Choosing university subjects in China. Br. J. Soc. Educ..

[21] Lareau A., Calarco J.M., Fiske S.T., Markus H.T. (2012). Class, cultural capital, and institutions: The case of families and schools. Facing Social Class: How Societal Rank Influences Interaction.

[22] Weininger E.B., Lareau A., Conley D. (2015). What money does not buy: Class resources and children’s participation in organized extracurricular activities. Soc. Forces.

[23] Dumais S.A. (2006). Early childhood cultural capital, parental habitus, and teachers’ perceptions. Poetics.

[24] Zhaoxin W., Binli C., Xiying W. (2023). The Manifestation and Decline of Academic Cultural Capital: The Educational Experience of the Rural-First Generation College Students in Elite Universities. Chongqing High. Educ. Res..

[25] Xiulan Y., Yan H. (2018). How to cultivate successful people from poor families—On the breakthrough of class restrictions from the cultural capital perspective. J. Higher Educ..

[26] Willis P. (2017). Learning to Labour: How Working Class Kids Get Working Class Jobs.

[27] Grayson P.J. (2011). Cultural capital and academic achievement of first generation domestic and international students in Canadian universities. Br. Educ. Res. J..

[28] Dong C., Ming Q. (2014). An examination of interaction inequality in classroom between teachers and students from the perspective of caring for the disadvantaged. Contemp. Educ. Sci..

[29] Gao K. (2021). The Influence of Family Cultural Capital on Academic Achievement of Rural Junior Middle School Students—Based on the Mediating Effect of Teacher-Student Interaction. Master’s Thesis.

[30] Li C. (2013). Discussion on the learning difficulties of left-behind children in rural areas—Based on the perspective of family cultural capital. Surv. Educ..

[31] Lareau A., Weininger E.B. (2003). Cultural capital in educational research: A critical assessment. Theory Soc..

[32] Bennett T., Frow J., Hage G., Noble G. (2013). Antipodean fields: Working with Bourdieu. J. Sociol..

[33] Mu G.M., Dooley K., Luke A. (2019). Bourdieu and Chinese Education.

[34] Mu G.M. (2020). Chinese education and Pierre Bourdieu: Power of reproduction and potential for change. Educ. Philos. Theory.

[35] Mu G.M., Mu G.M., Dooley K., Luke A. (2018). Resistance as a sociological process of resilience: Stories of under-resourced migrant families. Bourdieu and Chinese Education.

[36] Shi Z., Li C., Mu G.M., Dooley K., Luke A. (2018). Bourdieu’s sociological thinking and educational research in Mainland China. Bourdieu and Chinese Education.

[37] Jæger M.M., Møllegaard S. (2017). Cultural capital, teacher bias, and educational success: New evidence from monozygotic twins. Soc. Sci. Res..

[38] Guba E.G., Lincoln Y.S. (1994). Competing paradigms in qualitative research. Handbook Qual. Res..

[39] Parker C., Scott S., Geddes A. (2019). Snowball Sampling.

[40] Janghorban R., Roudsari R.L., Taghipour A. (2014). Skype interviewing: The new generation of online synchronous interview in qualitative research. Int. J. Qual. Stud. Health Well-Being.

[41] Braun V., Clarke V. (2006). Using thematic analysis in psychology. Qual. Res. Psychol..

[42] Merriam S.B. (1998). Qualitative Research and Case Study Applications in Education.

[43] Braun V., Clarke V. (2012). Thematic Analysis.

[44] Allsop D.B., Chelladurai J.M., Kimball E.R., Marks L.D., Hendricks J.J. (2022). Qualitative methods with Nvivo software: A practical guide for analyzing qualitative data. Psych.

[45] Xie X. (2010). Why are students quiet? Looking at the Chinese context and beyond. ELT J..

[46] Tang Q., Yu G. (2003). Investigation and analysis of the relationship between teachers and students in universities. Stat. Decis..

[47] Li J., Hu Y. (2017). The heterogeneity effect of teacher-student relationship on students’ achievement in different culture context: Based on PISA2012 data of China and USA. Henan Soc. Sci..

[48] Moore R. (2004). Cultural capital: Objective probability and the cultural arbitrary. Br. J. Sociol. Educ..

[49] Bourdieu P., Passeron J.C. (1990). Reproduction in Education, Society and Culture.

[50] Lareau A. (2015). Cultural knowledge and social inequality. Am. Sociol. Rev..

[51] DiMaggio P. (1982). Cultural capital and school success: The impact of status culture participation on the grades of US high school students. Am. Sociol. Rev..

[52] Aschaffenburg K., Maas I. (1997). Cultural and educational careers: The dynamics of social reproduction. Am. Soc. Rev..

[53] Cheng S.T., Kaplowitz S.A. (2016). Family economic status, cultural capital, and academic achievement: The case of Taiwan. Int. J. Educ. Dev..

