# Supplementary material for: The Roles of Cultural Capital in Teacher–Student Interactions in China: A Qualitative Study of Students in Higher Vocational Colleges
Source: Behav Sci (Basel). 2023 Aug 19;13(8):690. doi: 10.3390/bs13080690 (PMC10452010; doi:10.3390/bs13080690)
Supplement: Supplementary file 1 [file behavsci-13-00690-s001.zip › behavsci-2532813-supplementary.pdf]

Main interview questions

1. Could you please talk about your personality during your school period, such as primary school, middle school, and high school?
2. Could you please talk about your learning experience during your school period, regardless of whether it was a positive or a negative experience?
3. Do you have impressive learning memories from your school period with your teachers, friends, parents, schools, and so on?
4. Could you please describe your feelings about every school entrance exam, regarding your interesting or unforgettable memories?
5. What is the meaning of every learning period for you at primary school, middle school, or high school? Why do you have this kind of thought?

Notes: all interviews were conducted in Chinese to obtain the participants’ more precise responses.
